# A real-time electronic symptom monitoring system for patients after discharge following surgery: a pilot study in cancer-related surgery

**DOI:** 10.1186/s12885-020-07027-5

**Published:** 2020-06-10

**Authors:** H. S. Richards, J. M. Blazeby, A. Portal, R. Harding, T. Reed, T. Lander, K. A. Chalmers, R. Carter, R. Singhal, K. Absolom, G. Velikova, K. N. L. Avery

**Affiliations:** 1 Medical Research Council ConDuCT-II Hub for Trials Methodology Research, National Institute for Health Research Bristol Biomedical Research Centre, Bristol Centre for Surgical Research, Bristol Medical School, Population Health Sciences, University of Bristol, 39 Whatley Road, Bristol, BS8 2PS UK; 2 Division of Surgery, University Hospitals Bristol NHS Foundation Trust, Bristol, BS2 8HW UK; 3 Medical Research Council ConDuCT-II Hub for Trials Methodology Research, Bristol Centre for Surgical Research, Bristol Medical School, Population Health Sciences, University of Bristol, 39 Whatley Road, Bristol, BS8 2PS UK; 4 Section of Patient-Centred Outcomes Research, Leeds Institute of Medical Research at St James’s, University of Leeds, St James’s Hospital, Leeds, LS9 7TF UK; 5 Queen Elizabeth Hospital Birmingham, Mindelson Way, Edgbaston, Birmingham, B15 2WB UK

**Keywords:** Adverse events, Pilot studies, Patient-reported outcomes, Electronic health record, Internet, Cancer, gastrointestinal, Gastrointestinal surgical procedures, Self-management

## Abstract

**Background:**

Advances in peri-operative care of surgical oncology patients result in shorter hospital stays. Earlier discharge may bring benefits, but complications can occur while patients are recovering at home. Electronic patient-reported outcome (ePRO) systems may enhance remote, real-time symptom monitoring and detection of complications after hospital discharge, thereby improving patient safety and outcomes. Evidence of the effectiveness of ePRO systems in surgical oncology is lacking. This pilot study evaluated the feasibility of a real-time electronic symptom monitoring system for patients after discharge following cancer-related upper gastrointestinal surgery.

**Methods:**

A pilot study in two UK hospitals included patients who had undergone cancer-related upper gastrointestinal surgery. Participants completed the ePRO symptom-report at discharge, twice in the first week and weekly post-discharge. Symptom-report completeness, system actions, barriers to using the ePRO system and technical performance were examined. The ePRO surgery system is an online symptom-report that allows clinicians to view patient symptom-reports within hospital electronic health records and was developed as part of the eRAPID project. Clinically derived algorithms provide patients with tailored self-management advice, prompts to contact a clinician or automated clinician alerts depending on symptom severity. Interviews with participants and clinicians determined the acceptability of the ePRO system to support patients and their clinical management during recovery.

**Results:**

Ninety-one patients were approached, of which 40 consented to participate (27 male, mean age 64 years). Symptom-report response rates were high (range 63–100%). Of 197 ePRO completions analysed, 76 (39%) triggered self-management advice, 72 (36%) trigged advice to contact a clinician, 9 (5%) triggered a clinician alert and 40 (20%) did not require advice. Participants found the ePRO system reassuring, providing timely information and advice relevant to supporting their recovery. Clinicians regarded the system as a useful adjunct to usual care, by signposting patients to seek appropriate help and enhancing their understanding of patients’ experiences during recovery.

**Conclusion:**

Use of the ePRO system for the real-time, remote monitoring of symptoms in patients recovering from cancer-related upper gastrointestinal surgery is feasible and acceptable. A definitive randomised controlled trial is needed to evaluate the impact of the system on patients’ wellbeing after hospital discharge.

## Background

Major abdominal surgery for upper gastrointestinal (UGI) cancer is a significant life event with a hospital stay of usually more than a week and a high risk of complications. For example, up to 5% of patients die within 30 days of surgical resection for oesophageal cancer. As many as 30–45% of UGI, hepatobiliary and cancer surgery patients require community care or experience complications post-discharge [1–3], including wound infections, sepsis and respiratory failure [4–7] requiring hospital treatment. Recovery from surgery for these procedures often takes many months. Symptoms such as fatigue, nausea and pain are frequent and severe [2], and patients report significant deficits to many aspects of their health-related quality of life (HRQL), including marked reductions in physical, social and role function [8, 9].

Advances in Enhanced Recovery After Surgery (ERAS) protocols mean that patients are increasingly discharged from hospital sooner. While earlier discharge may be feasible and safe [10–12], shorter hospital stays are associated with reductions in the provision of patient information and symptom self-management advice [13]. As a result, patients recovering at home may experience unsatisfactory pain management and increased anxiety [13]. Furthermore, ERAS protocols are not standardised and do not routinely encompass the post-discharge recovery period, meaning that follow-up care is often fragmented [13, 14]. Patients may be followed-up through telephone calls from nurse specialists, but this practice is likely to vary between hospitals. Once at home, detection of problems and complications relies on the patient’s ability to distinguish between expected and concerning symptoms and to access appropriate clinical services. However, patients may be uncertain about when to contact a health professional outside of routine clinical appointments, which can delay them seeking help [15, 16]. Late detection of complications after discharge can lead to poor outcomes, impaired HRQL and increased emergency department admissions [17].

Incorporating patient-reported outcome (PRO) measures into routine clinical practice has been shown to enhance symptom monitoring and the detection of complications in cancer patients [18, 19]. Electronic platforms to collect PROs (ePRO) are an efficient means of capturing PRO data after patients have left hospital. Specifically, ePRO systems that use clinically-derived algorithms to interpret patient-reported symptom data in the context of their clinical characteristics (e.g. disease stage, surgical procedure, recovery pathway) may provide patients with pertinent self-management advice to support their recovery [20–23]. For optimal performance, full integration of ePROs with electronic hospital records (EHR) and clinical information systems can also provide clinicians with access to ‘real-time’ ePRO data. This enables prompt clinical intervention [24, 25] and allows clinicians to consider patient-reported symptoms alongside other clinical data to optimally plan appropriate care. This has been shown to enhance quality of care and communication [26, 27] and improve patients’ satisfaction with care [18].

Several ePRO systems, developed for patients undergoing chemotherapy [22, 28–32], have been shown to improve HRQL [22, 29–31, 33] and survival [29]. Few studies, however, have evaluated the effectiveness of ePRO systems in patients undergoing surgery for cancer. The aim of this pilot study was to examine the feasibility of a full-scale definitive randomised controlled trial (RCT) designed to evaluate the impact of a real-time, remote electronic monitoring (ePRO) system on patients’ wellbeing after discharge following cancer-related UGI surgery.

Specific study objectives were to: (i) explore participant eligibility and recruitment; (ii) examine participant ePRO symptom-report response rates and data completeness; (iii) examine the frequency of patient-reported symptoms and ePRO system actions; (iv) explore patient and clinician perspectives on using the ePRO system; (v) evaluate the technical performance of the ePRO system; (vi) pilot potential outcome measures for use in a future main trial.

## Methods

### Development of the ePRO surgery system

The ePRO surgery system was developed initially in the example context of cancer-related UGI surgery. The system was developed in close collaboration with multiple key stakeholder groups, including patients, patient representatives and health care professionals (HCPs) (i.e. cancer nurse specialists (CNS), dietitians and surgeons) responsible for routine’ post-discharge care. The development of the ePRO system and the electronic hosting platform has been described in full elsewhere [34–36]. The IT elements, developed in the eRAPID study [36], include a patient website with secure login function, web-based symptom-report questionnaire software (QTool) and a web application interface for secure transfer of data to EHR, enabling clinicians to view symptom-reports. Clinically-derived algorithms were programmed into the self-report scoring system, allowing severity specific tailored self-management advice to be provided to patients and email notifications sent to nominated clinicians.

### ePRO system clinically derived algorithms to guide patient management by symptom severity

The system uses clinically derived algorithms to stratify patients’ ePRO symptom-report responses into three levels of symptom severity with each triggering a different ‘level’ of action within the ePRO system (Table 1), and is described in detail elsewhere [34]. For symptoms indicative of a complication, an email alert is sent to the CNS team. For potentially concerning symptoms, participants are advised to call an HCP. For expected symptoms, the ePRO system provides participants with self-management advice. The ePRO symptom-report comprised 35 questions selected from validated European Organisation for Research and Treatment of Cancer (EORTC) questionnaires. Items were selected for their relevance to symptoms and complications experienced by patients after cancer-related UGI surgery (including oesophageal, gastric and hepato-pancreato biliary cancer) [34] and focus on severity in the preceding week (e.g. During the past week, have you had pain? Possible responses are: not at all; a little; quite a bit or very much). Symptom domains included EORTC domains physical function, nausea, vomiting, eating and digestive problems, jaundice, fever and pain [34]. Graphs displaying scores for each symptom are produced within the ePRO system, enabling patients and clinicians to monitor symptom occurrence and severity over the course of patients’ recovery. Following their development, refinement and testing of the algorithms triggering the ePRO system feedback and alerts was also undertaken as part of earlier work [34]. Specifically, questionnaire data from 27 participants (18 male, mean age 63 years) who reported clinically significant symptoms was compared, firstly, with advice provided by a CNS or study research nurse during audio-recorded routine telephone consultations and weekly telephone interviews and, secondly, with any subsequent clinical events or outcomes of participants (e.g. such as re-intervention, re-admission to hospital, visit to GP or primary healthcare providers). The latter were identified from hospital readmission alerts, hospital EHR, and patients’ reports of accessing healthcare services reported during weekly follow-up telephone interviews. Refinement and iteration of the algorithms continued until it was considered that no further changes were required.

**Table 1:** Guided patient management by symptom severity within the ePRO system

**Symptom severity level**	**ePRO system action/advice**	**Example of ePRO action/advice for shortness of breath**
Level 0: minimal/no symptoms	No patient advice required	Thank you for completing the questionnaire.
Level 1: expected symptom(s)	Patient advice: self-management advice	Some shortness of breath after physical activity such as climbing the stairs is a normal part of recovery. You may wish to consider the advice below…
Level 2: potentially concerning symptom(s)	Patient advice: contact a healthcare professional today if symptom is new or unreported	If you have not already discussed your shortness of breath with your medical team we recommend that you contact your CNS team today to discuss your symptoms
Level 3: symptom(s) indicative of a complication	(i) Patient advice: contact a healthcare professional immediately (ii) Clinician alert: automated email to a Cancer Nurse Specialist	We recommend that you contact the hospital now to discuss your symptoms with the medical team. If you are unable to contact the CNS team, please call your GP to discuss your symptoms today

### Design and setting

This mixed methods prospective pilot study was conducted at two National Health Service (NHS) hospital trusts in England. Feasibility of the fully functional ePRO system accessible via EHRs was examined at Bristol Royal Infirmary, University Hospitals Bristol NHS Foundation Trust (Centre 1). Integration of electronic monitoring systems into hospital EHRs can be problematic and the extent of integration variable. As such, the feasibility and applicability of the system at a second site (Heartlands Hospital, University Hospitals Birmingham, Centre 2) where integration was not possible was explored. In the version of the ePRO system tested at Centre 2, clinicians could not access patients’ symptom-reports via EHRs. Data from Centre 2 were therefore analysed separately, with a focus on examining participant recruitment, ePRO symptom-report response rates and data completeness. Consecutive patients at both centres were recruited and invited to complete online symptom-reports at the point of hospital discharge, twice in the first week post-discharge and weekly for 8 weeks thereafter. Recruitment rates, online symptom-report completion rates and activation of clinical algorithms were monitored. Patients were interviewed weekly and additional outcomes related to health status were collected on paper. The CNS teams at both centres received Level 3 alerts via automatic emails, and in Centre 1 the lead CNS was interviewed weekly.

### Participant sampling and recruitment

Consecutive patients who had undergone cancer-related UGI surgery (including oesophageal, gastric or hepato-pancreato biliary cancer) at Centre 1 between August 2017 and March 2018 and Centre 2 between November 2017 and October 2018 were screened for study eligibility by a hospital research nurse reviewing inpatient clinic lists. Patients were considered eligible if they had undergone cancer-related UGI surgery, were ready for hospital discharge to their home, had access to a computer/mobile device and the internet at home, were 18 or over and were fluent in English. Patients were approached for recruitment by a research nurse immediately prior to hospital discharge (e.g. while on the hospital ward). Eligible patients were given a participant information leaflet and the opportunity to ask questions, and those wishing to participate were asked to provide written informed consent. Demographic and clinical details were recorded. Participants were provided with unique login details and use of the ePRO system was demonstrated by the research nurse, during which participants completed the baseline (pre-discharge) ePRO symptom-report and paper version of the additional measures (e.g. EQ-5D and Fact-G).

### Data collection

Recruitment, response rates and data completeness were recorded. An overview of data collected at assessment timepoints at each site is provided in Table 2.

**Table 2:** Data collection at baseline and post-discharge assessment timepoints

	**Point of discharge**	**Post-discharge**										
	**Baseline**	**2–3 days**	**5–7 days**	**Week 2**	**Week 3**	**Week 4**	**Week 5**	**Week 6**	**Week 7**	**Week 8**	**Post-study**	**End of study interviews**
Screening log completion (Centre 1 only)	✓											
Participant demographic and clinical characteristics	✓											
ePRO questionnaire completion	✓	✓	✓	✓	✓	✓	✓	✓	✓	✓		
FACT-G & EQ-5D questionnaires^a^	✓				✓				✓			
Weekly follow-up participant interviews	✓	✓	✓	✓	✓	✓	✓	✓	✓	✓		
Health resource use questionnaire^b^											✓	
End of study participant interviews (*10% participants*)												✓
Weekly follow-up clinician interviews (Centre 1 only)		✓	✓	✓	✓	✓	✓	✓	✓	✓		
End of study clinician interviews (Centre 1 only)												✓

*Abbreviations*: *ePRO* electronic patient-reported outcome, *FACT-G* Functional Assessment in Cancer Therapy Scale – General

^a^The FACT-G is a standardised cancer specific health related quality of life measure. The EQ-5D is a standardised measure of health status used in clinical and economic evaluation. These measures were administered in paper format

^b^The health resource use questionnaire included items to record use of prescription and non-prescription medication and other costs associated with patients’ recovery from surgery. These measures were administered in paper format

#### Participant eligibility and recruitment

Screening logs were completed to examine the number of participants eligible, approached, recruited and withdrawn from the study. Rates and reasons for non-eligibility, declining participation and participant withdrawal were monitored.

#### ePRO symptom-report response rates and data completeness

Participants were instructed to complete the online ePRO symptom-report twice in the first week post-discharge (day two-three and day five-seven) and then weekly for 8 weeks. These timepoints were selected to reflect clinically relevant timepoints for monitoring of patients during their recovery post-discharge following UGI surgery [34]. However, participants were told that they could complete the symptom-report at additional timepoints if they wished (e.g. if they experienced new symptoms). Automated reminders (by text message and/or email depending on participants’ preferences) were sent when participants were due to complete the ePRO symptom-report.

#### Frequency of reported symptoms and ePRO system actions

In order to determine the suitability of the actions and advice generated by the ePRO system, the frequency, severity and type of reported symptoms and actions generated at each timepoint were examined.

#### Patient and clinician perspectives

##### Participant interviews

Members of the research team (AP, HR, MS) conducted weekly semi-structured telephone interviews with participants to coincide with ePRO symptom-report timepoints, generally shortly after questionnaire completion. Interviews focused on participants’ views of using the ePRO system and any responses they had had to ePRO system actions. For example, if a participant reported a potentially concerning symptom that had generated a Level 2 action, the patient was asked whether they had contacted an HCP as advised, and any subsequent outcomes resulting from such HCP contact were documented. The purpose of the weekly and end of study interviews was not to discuss patients’ symptoms or provide advice regarding managing or seeking treatment for problematic symptoms. Instead, the interviews were undertaken to examine participants’ perspectives on the usefulness of the ePRO system and feedback from the perspective of examining the feasibility and usability of the system. The interview guides were adapted from pilot work to develop the ePRO system [34] and are provided in Additional file 1. Reasons for not adhering to ePRO system actions/advice were also recorded. Participants generating Level 1 self-management advice for expected symptoms were asked for their views on the advice and its relevance and usefulness. A purposive sample of approximately 10%, to include patients who reported a range of clinical symptoms, were also interviewed at the end of the study to explore their experiences of using the ePRO system. All interviews were audio-recorded.

##### Hospital readmissions

Clinical data were collected from hospital EHR for Centre 1 participants who had been readmitted to hospital with complications during their recovery. These data were compared with interview data to establish whether participants had completed a corresponding symptom-report and any actions that may have been generated.

##### Clinician interviews

The lead CNS in Centre 1 was interviewed weekly by telephone by a study researcher (HR) to determine the frequency, nature and relevance of any clinical contact they had received from participants as a direct result of ePRO system actions. End-of-study interviews were also conducted with the lead CNS and the hospital dietitian responsible for the care of participants. These explored the practicality and usefulness of the ePRO system in the context of routine clinical care.

#### Technical performance

Technical functionality of the ePRO system was monitored throughout the study, including integration with EHR, failed logins and delivery of email clinician alerts and participant text/email reminders.

#### Piloting of potential outcome measures for a main trial

Participants were mailed paper copies of the EuroQol EQ-5D-5 L [37] and Functional Assessment of Cancer Therapy – General (FACT-G) [38] questionnaires to complete at baseline (pre-discharge) and at weeks four and eight post-discharge. The EQ-5D-5 L is a standardised, validated measure of health status that is used in the clinical and economic evaluation of healthcare and population health surveys. It provides a single index value and descriptive profile that contributes to a health economic evaluation across five dimensions: mobility, self-care, usual activities, pain/discomfort and anxiety/depression. The five response levels range from ‘no problems’ to ‘extreme problems’. The EQ-5D also includes a visual analogue scale relating to subjective overall health status. The FACT-G is a cancer specific measure that is widely used in research studies, measuring four domains of HRQL: physical wellbeing, social or family wellbeing, emotional wellbeing and functional wellbeing. Cumulative scores range from 0 to 108 with higher scores indicating better HRQL. Questionnaire response rates and data completeness were examined to explore their feasibility for use as potential outcome measures in a future RCT.

Methods for collecting health resource use data were explored. Patients were asked to complete a health resource use questionnaire [35] at the end of the study (9 weeks post-discharge). The questionnaire included items to record use of prescription and non-prescription medication and other costs associated with patients’ recovery from surgery. Patients were also contacted weekly by the study researcher to record the frequency and reasons for contact with HCPs (e.g. general practitioner (GP), community nurse, etc).

### Data analyses

Summary descriptive statistics were used to examine participant screening, recruitment, demographic and clinical characteristics. Symptom-report response rates, data completeness and reasons for non-completion were summarised. Descriptive statistics also summarised symptom-report response rates and data completeness, frequency and severity of symptoms reported and any actions generated. Audio-recordings of interviews with participants and clinicians were listened to by a researcher (HR) and those containing data of relevance to the study objectives were transcribed verbatim (targeted transcription). Textual data were analysed in accordance with the principles of thematic analysis [39], in which codes were generated and applied to sections of text. Codes were reviewed and refined through discussion with the study team. Thematic analysis is a widely-used methods of qualitative analysis involving the identification and interpretation of underlying themes and concepts within the wider context of the data. The interview topic guide was iterated following discussion with the study team as data collection and participant recruitment proceeded. Analysis was conducted until thematic saturation was reached, whereby existing themes ceased to evolve and no new themes were identified [40]. Here we report the main themes within the context of a wider mixed methods study.

## Results

### Participant eligibility and recruitment

**Fig. 1:**
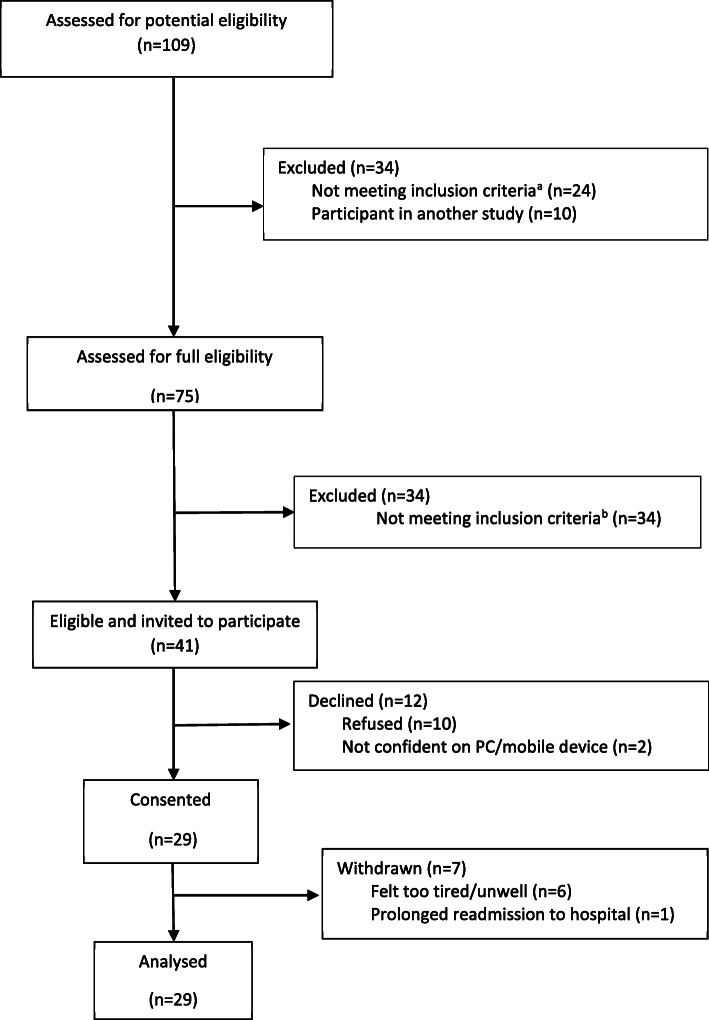
Recruitment details for Centre 1 Bristol participants. ^a^ including: not undergoing planned procedure (*n* = 17), discharged home unexpectedly early or not discharged to home (*n* = 4), missed due to administration errors (*n* = 2), patient was under 18 (*n* = 1). ^b^ including: no home access to a PC/internet (*n* = 15), discharged home unexpectedly early or not discharged to home (*n* = 11), not fluent in English (*n* = 3), missed due to administration errors (*n* = 2), unable to comply with follow up (*n* = 3)

One hundred and nine patients were screened for eligibility at Centre 1, of which 41 (38%) were eligible and invited to participate, and 29 (71%) consented. (Fig. 1). Patients who agreed to take part were similar to those who declined in terms of demographics (participants: 66% male, refusers: 50% male) or age (participants: mean age 64, range 43–81 years, refusers: mean age 59, range 30–74 years). Reasons for ineligibility included participants not undergoing their planned surgical procedure (17, 25%), having no home access to a computer/mobile device or internet (15, 22%) (Fig. 1). Seven (24%) Centre 1 participants withdrew from the study due to feeling too tired or unwell or a prolonged re-admission to hospital. Approximately half (*n* = 15, 52%) of patients had undergone oesophago-gastric resection procedures, with an average hospital stay of 12 days. Participant demographic details are provided in Table 3. In Centre 2, 20 patients were screened for eligibility, of which 11 consented to participate. All 11 participants had undergone oesophago-gastric resection procedures (Table 3). Two (18%) Centre 2 participants withdrew from the study.

**Table 3:** Participant baseline clinical and demographic details

	**Centre 1** **Bristol participants (*n*** **= 29)**	**Centre 2** **Birmingham participants (*n*** **= 11)**
**Sex, n (%)**		
Male	19 (66)	8 (73)
**Age, years**		
Mean (SD)^a^	64.2 (9.8)	63.4 (14.9)
Range	43–81	42–81
**Ethnicity, n (%)**		
White British	22 (76)	11 (100)
Chinese	1 (3)	0
Not stated	6 (21)	0
**Cancer diagnosis, n (%)**		
Yes	21 (72)	11 (100)
**Length of hospital stay, days**		
Mean (SD)^a^	12 (9)	18 (12)
Median (IQR)^b^	9 (6–14)	11 (10–15)
Range	3–35	9–45
**Surgical procedure received, n (%)**		
Oesophago-gastric resection	15 (52)	11 (100)
Hepatobiliary resection	14 (48)	0
**Marital status, n (%)**		
Married/civil partnership/cohabiting	26 (91)	5 (46)
Single	1 (3)	1 (9)
Divorced/separated	1 (3)	2 (18)
Widowed	1 (3)	3 (27)
**Education, n (%)**		
Further education	22 (76)	6 (55)
Degree/professional qualification	15 (52)	5 (46)
**Employment status, n (%)**		
Retired	15 (52)	6 (55)
Working full-time	6 (20)	3 (27)
Working part-time	4 (14)	1 (9)
Not in paid employment	4 (14)	1 (9)
**Computer usage, n (%)**		
Daily	26 (90)	8 (73)
Weekly	3 (10)	1 (9)
Rarely	0	2 (18)
**Proficiency with computer, n (%)**		
Easy	21 (72)	7 (64)
Sometimes difficult	8 (28)	2 (18)
Difficult	0	2 (18)

^a^Standard deviation

^b^Interquartile range

### ePRO symptom-report response rates and data completeness

Response rates of active participants (i.e. participants who had not withdrawn from the study) in Centre 1 for the ePRO symptom-report exceeded 70% at all timepoints (range 72–93%), excluding day two-three post-discharge (55%). Between 30/08/2017 and 17/04/2018, 29 participants completed the ePRO symptom-report a total of 197 times (median 9, range = 1–11). Most common reported reasons for non-completion included participants starting chemotherapy (12%) and not wanting to complete the symptom-report at that timepoint (10%) (see Table 4). In Centre 2, ePRO symptom-report response rates exceeded 60% at all timepoints (range 63–100%), except for week eight (50%). Between 10/11/2017 and 22/11/2018, participants completed the ePRO symptom-report a total of 63 times (median 7, range 1–10). Reasons for non-completion were not recorded.

**Table 4:** Response rates and reasons for non-completion of ePRO questionnaire

	**Baseline**^**a**^	**2–3 days**	**5–7 days**	**Week 2**	**Week 3**	**Week 4**	**Week 5**	**Week 6**	**Week 7**	**Week 8**	
Active participants^b^, n	29	29	29	27	25	24	24	23	22	22	
Active participants completing ePRO questionnaire, n (%)^c^	27 (93)	16 (55)	23 (79)	20 (74)	21 (84)	21 (88)	22 (92)	19 (83)	16 (72)	17 (77)	
Active participants not completing ePRO questionnaire, n	2	13	6	7	4	3	2	4	6	5	
**Reasons for non-completion**:											**Totals**
Withdrawn from the study	0	0	2 (33)	2 (29)	1 (4)	0	1 (50)	1 (25)	0	0	7
Unknown - participant could not be reached for weekly phone interview	0	7 (54)	4 (67)	2 (29)	0	2 (67)	1 (50)	2 (50)	2 (33)	1 (20)	21
Did not want to	1 (50)	1 (8)	0	1 (14)	1 (4)	0	0	0	0	1 (20)	5
Started chemotherapy^d^	0	0	0	0	0	0	0	1 (25)	2 (33)	3 (60)	6
Admin failure (e.g. overlap of dates/ University closure)	1 (50)	1 (8)	0	0	1 (4)	1 (33)	0	0	1 (17)	0	5
Re-admitted to hospital	0	1 (8)	0	2 (29)	0	0	0	0	0	0	3
Too busy	0	1 (8)	0	0	1 (4)	0	0	0	1 (17)	0	3
Too unwell	0	2 (15)	0	0	0	0	0	0	0	0	2
Total number of ePRO questionnaire completions	**19**^**e**^	**18**	**23**	**21**^**f**^	**21**	**21**	**22**	**19**	**16**	**17**	**197**

^a^Baseline completion takes place at the point of hospital discharge. All subsequent timepoints are length of time since hospital discharge

^b^ Participants who had not withdrawn from the study

^c^The design of the ePRO system ensures all items are completed, except for completions that were abandoned prior to submission

^d^ Patients halted completion of ePRO if they commenced chemotherapy during follow-up

^e^ 8 baseline completions triggering a Level 3 action were removed from the dataset post-hoc because they were later determined to be clinically irrelevant by participants and clinicians (as they were retrospectively reporting symptoms experienced immediately post-surgery that had resolved). This will inform the next iteration of algorithms to be using in a full RCT

^f^ The ePRO system allows multiple completions at each timepoint

### Frequency of reported symptoms and ePRO system actions

**Fig. 2:**
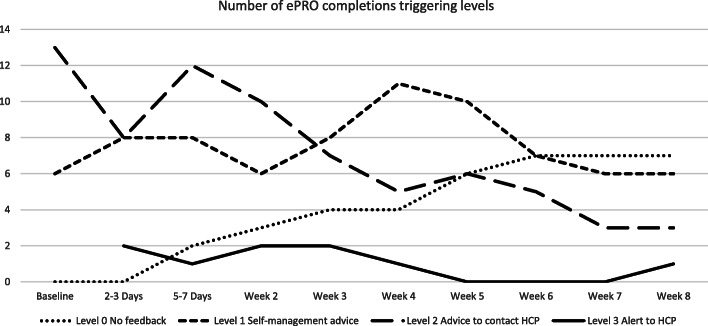
Total levels of feedback generated by timepoint

Frequencies of symptoms reported by Centre 1 participants and associated actions triggered by the ePRO system are shown in Table 5. Of the nine Level 3 email alerts to clinicians, seven (78%) were generated in the first 3 weeks following discharge from hospital (Fig. 2). Over half (*n* = 43, 60%) of the 72 Level 2 actions (participant advice to call an HCP) were triggered within the first 2 weeks post-discharge, with a further 15% (*n* = 11) triggered after 6 weeks post-discharge. Most (*n* = 48, 63%) of the 76 Level 1 actions (self-management advice) were triggered after 3 weeks post-discharge, the majority of which were triggered at weeks four and five. Of the 40 forty Level 0 feedback (no participant advice required), 27 (68%) instances were triggered after 5 weeks post-discharge.

**Table 5:** Frequency of reported symptoms and ePRO system actions by patients at Centre 1 (*n* = 29)

**ePRO system reported symptoms and actions.**	**Number of times (%) symptom triggered action (*n*** **= 197)**	**Number (%) of patients triggering actions at any timepoints (*n*** **= 29)**
**Level 3 action – alert to CNS**^**a**^	**9 (4.6)**	**3 (10.3)**
Pain	7 (3.6)	2 (6.9)
Fever and chills	2 (1.0)	1 (3.4)
**Level 2 action - advice to contact HCP**^**a**^	**72 (36.5)**	**24 (82.8)**
Wound problems	32 (16.2)	14 (48.3)
Appetite loss	26 (13.2)	12 (41.3)
Fever and chills	18 (9.1)	6 (20.7)
Physical function	10 (5.1)	8 (27.6)
Nausea and vomiting	8 (4.1)	8 (27.6)
Shortness of breath	7 (3.6)	5 (17.2)
**Level 1 action - symptom advice**^**b**^	**76 (38.6)**	**22 (75.9)**
Fatigue	58 (29.4)	20 (70.0)
Pain	27 (13.7)	12 (41.3)
Physical function	22 (11.2)	10 (34.5)
Constipation	20 (10.2)	10 (34.5)
Nausea and vomiting	20 (10.2)	8 (27.6)
Reflux	17 (8.6)	8 (27.6)
**Level 0 (minimal/no symptoms) - No advice required**	**40 (20.3)**	**9 (31.0)**

^a^ Level 2 and 3 actions can be triggered by the reporting of multiple symptoms

^b^ If more than 6 symptoms reached Level 1 threshold, only the top 6 symptoms (ranked a priori by healthcare professionals) were listed with symptom-specific advice at completion of ePRO

An additional eight Level 3 alerts were generated from the baseline data completion prior to patients being discharged. These data were removed from the dataset post-hoc following consultation with clinicians, as they were deemed irrelevant due to patients still being in hospital.

In Centre 2 one Level 3 alert was triggered at week three, and 14 (70%) of the 20 Level 2 actions were triggered in the first 3 weeks post-discharge. Most (*n* = 25, 69%) of the 36 Level 1 self-management advice were triggered after 3 weeks post-discharge, while five (83%) of the six Level 0 feedback (83%) were triggered after 4 weeks post-discharge.

#### Symptoms triggering level 3 actions (alert to CNS)

Level 3 alerts (automated email to CNS team) were triggered nine times (4.6% of all completions) by a total of three (10%) participants in Centre 1 (see Table 5). Telephone interviews with participants demonstrated that when they made direct contact with the CNS team as instructed by the system they reported positively on their experience:

*PT 1237: “It was great. It was reassuring…And it’s what I need actually…I can’t just phone my GP…I’m not even on my own GP’s list because I’ve been transferred [to a different geographical region] temporarily.”*

Most Level 3 alerts (*n* = 7, 78%) were generated for pain symptoms. Six of the seven pain alerts were from a single participant due to a pre-existing chronic pain condition, and were therefore not related to recovery from surgery. Weekly telephone interviews revealed that no action was taken by the CNS team relating to these alerts, as the participant was already receiving appropriate treatment from another care team.

The remaining two non-pain related Level 3 alerts were generated by a single participant reporting symptoms of fever and chills due to a complication following surgery. Although an alert was generated, no further action was taken by the participant or the CNS team, in accordance with Level 3 advice because these symptoms had already been treated prior to ePRO system completion:

*PT 1184: “Because I’ve been completing the form on a Friday, if there’s been any circumstances where I’ve needed to contact someone it’s probably been earlier in the week and action’s already been taken…We were already dealing with [fever symptoms]. Probably already [contacted HCP].”*

This demonstrates the need for a further minor refinement to the Level 3 and 2 algorithms to ensure that erroneous symptoms relating to underlying health conditions and relevant symptoms that are already being well-managed do not trigger alerts to clinicians.

#### Symptoms triggering level 2 actions (advice to contact an HCP)

**Fig. 3:**
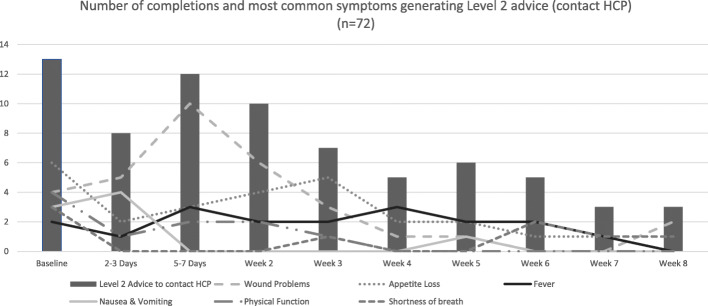
Frequency of Level 2 Advice generated by timepoint. ^1^Higher scores indicate worse symptoms. ^2^Higher scores in physical function indicate better physical function

In total, 72 Level 2 actions (advice to contact an HCP) were triggered by 24 (83%) participants in Centre 1 and were most frequently related to wound problems (44%) and appetite loss (36%). Additional symptoms generating Level 2 advice included fever (24%), physical function (13%) and nausea or vomiting (11%). Figure 3 illustrates the distribution of the most frequent symptoms generating Level 2 advice by recovery timepoint.

Of the 72 Level 2 actions triggered, corresponding weekly telephone interview data were available for 36 (50%) of these events from 17 participants.

##### Adherence to level 2 action advice to contact an HCP

In most instances (23/36, 64%), participants adhered to the Level 2 advice to only contact an HCP if their symptoms were new or previously unreported. In eight (35%) cases, all participants with new symptoms followed advice to contact an HCP, of which four participants contacted the CNS team and four contacted a GP. Contacting an HCP resulted in four participants receiving advice and/or reassurance about their symptoms, three undergoing clinical investigations or interventions (e.g. blood tests, appointments), and one receiving a prescription for antibiotics for a wound infection:

*PT 1213: “The second time [called GP following Level 2 advice] I was feeling so tired and [GP] sent me for a blood test and found I’m low on something and I’m on four tablets a day now.”*

In 15 cases (65%), participants appropriately followed advice not to contact an HCP because their symptoms were already being appropriately managed and/or they had upcoming clinical appointments:

*PT 1205: “I was already in contact with people about [symptoms], so the questionnaire told me to do that anyway, but I was already doing it. Had I not been doing it, it would have helped me, but I was doing it anyway.”*

Participants described how receiving advice to contact an HCP was reassuring, as until that point they had been uncertain whether their symptoms required clinical input or further intervention:

*PT 1242: “I’ve been thinking, should I ring the nurse about constipation or should I not?...Sometimes you feel you shouldn’t ring your nurse, you know. Silly really because that’s what the nurse is there for isn’t it.”*

##### Non-adherence to level 2 action advice to contact an HCP

Of the 13 (36%) instances when participants did not adhere to Level 2 advice, reasons for not contacting an HCP included feeling that their symptoms did not warrant reporting (*n* = 6), for example, because they already had an HCP appointment scheduled:

*PT 1224: “It [ePRO] said that I’ve been having problems with my wound, and it said that I ought to see somebody within 48 hours. Well as I had an appointment…this morning at [the hospital] I didn’t see any need to call anybody.”*

Other reasons included participants forgetting that they had received the Level 2 advice (*n* = 6, 46%), or stating an unwillingness to contact an HCP (*n* = 1).

#### Symptoms triggering level 1 symptom (self-management advice)

**Fig. 4:**
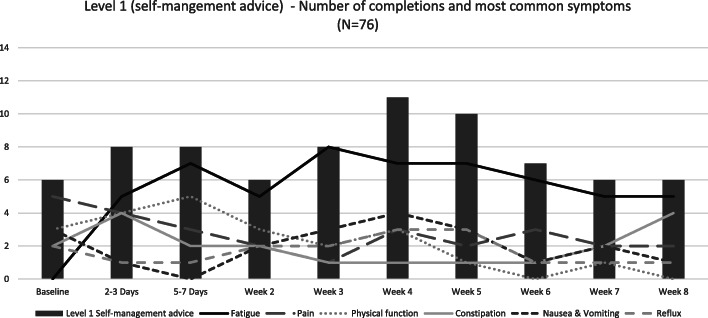
Frequency of Level 1 self-management advice generated by timepoint. ^1^Higher scores indicate worse symptoms. ^2^Higher scores in physical function indicate better physical function

Level 1 self-management advice was triggered a total of 76 times (39% of completions) by 22 (76%) participants in Centre 1 post-discharge. Typically, advice was provided for three symptoms (median = 2.5, range = 1–6) per Level 1 action triggered. Advice was generated most frequently for fatigue (*n* = 58) and/or pain (*n* = 27). Additional symptoms generating Level 1 advice included physical function (*n* = 22), nausea or vomiting (*n* = 20) and constipation (*n* = 20). Figure 4 illustrates the frequency and distribution of the six most frequent symptoms triggering Level 1 advice at each timepoint.

#### Level 0 feedback for minimal symptoms (no action required)

Level 0 feedback was generated a total of 40 times (20% of completions) by approximately one third of participants in Centre 1 (*n* = 9, 31%) (Fig. 2).

#### Hospital re-admissions

Five participants were re-admitted to hospital during the study for complications relating to fevers/infections (*n* = 3), nausea & vomiting (*n* = 1) and bile leak (*n* = 1). These participants completed the ePRO symptom-report a total of 14 times within 7 days of re-admissions. These completions resulted in two Level 3 events (14%), nine Level 2 events (64%) and one Level 1 event (7%), indicating that, in the majority of instances, the ePRO system produced appropriate advice based on complication-related symptoms reported by patients. While it is not possible to provide exact numbers, there were several instances where participants were re-admitted to hospital for complications related to post-operative infections without having completed the ePRO system. In these instances, participants recognised the severity of their symptoms and contacted the care team without the need for prompting from voluntary completion of the ePRO system.

### Patient and clinician perspectives

#### Participant interviews

One hundred and nine (63%) of a potential 173 weekly telephone interviews were completed. All 29 participants were interviewed at least once following discharge. The main reasons for non-completion were participants being unavailable or non-contactable. The key themes identified from targeted transcriptions of 35 weekly telephone interviews and three end-of-study interviews with 16 participants are reported below.

##### Reassurance from ePRO system advice

Most participants reported positively on their experience of using the ePRO system. Participants described how they found the Level 1 symptom self-management advice to be valuable because it provided reassurance about how to manage their symptoms while at home. Participants cited the benefit of receiving confirmation that their symptoms were typical for their stage of recovery as the main reason for finding the ePRO system reassuring:

*PT 1230: “It’s all about a bit of reassurance really. The minute you get a bit of reassurance, everything else seems a bit easier.”*

*PT 1242: “The system’s good at telling you what’s normal, or not even normal, but that other people have this [experience of symptoms].”*

Additionally, participants valued being provided with new symptom management advice to help them better manage their own symptoms:

*PT 1182: “Things like not drinking too much coffee tea and alcohol, which I hadn’t thought of before, I didn’t think that would affect the issue but apparently it does, so I’ll abide by that advice.”*

Some participants acknowledged that the advice provided by the ePRO system was similar to advice they had originally received from their care teams. Participants found the ePRO system advice useful for reminding them to follow advice they may have forgotten and reinforced the guidance they had received from HCPs:

*PT 1226: “[The same advice] had been mentioned earlier on in the process, I think when I had my initial consultation…and it just reminded me of all those things…And I found them very useful, although they weren’t adding anything new, again they were reassuring.”*

Participants also stated that the reassurance they gained from the ePRO system contributed to overcoming feelings of isolation and uncertainty following discharge from hospital:

*PT 1188: “I mean you get monitored so much in the hospital…And then you come out and there’s nothing at all. It’s like a sudden drop off a cliff really. And your first week and that you go well, am I OK? You know they’ve been checking for all this time.”*

Participants discussed the graphs produced by the ePRO system, which displayed their individual symptom-report scores over the course of the study and described these as useful for tracking their progress over time. Participants spoke about the reassurance they gained from being able to objectively see that their symptoms were improving:

*PT 1205: “It was telling me that I was getting better I think, which is what I was hoping for. That was quite informative I thought, looking at the graphs at the end, that was quite good.”*

##### Relevance of ePRO advice

Participants discussed ePRO advice as being relevant to their symptoms and addressing their concerns about symptom management. For many participants the advice generated accurately reflected their symptom experiences:

*PT 1182: “I think it covered the two main [symptoms] that I was bothered about.”*

Participants described the benefit of receiving relevant symptom self-management advice tailored specifically to them and found this helpful:

*PT 1208: “At the end you get to read up about what the suggestions are to cope with the different things I might have raised and that’s always very helpful…and aimed specifically at me.”*

#### Clinician interviews

The majority of ePRO-initiated clinician contact (e.g. contact from participants following triggering of a Level 2 action or clinician alert following a Level 3 action) was considered timely and appropriate, and clinicians reported that they did not think any concerning symptoms had been ‘missed’ by participants who received ePRO advice to contact them*.* In some cases, these directly informed the clinical management of patients. On several occasions, for example, clinicians’ arranged GP or hospital appointments for patients who had been advised to call them. The majority of ePRO-initiated clinician contact (e.g. contact from participants following triggering of a Level 2 action or clinician alert following a Level 3 action) was considered timely and appropriate:

*Clinician 1: “I’m not sure he would have thought to ring us, as his first port of call. And we were able to sort of triage what the problem was and make sure he spoke to the right person.”*

Similar to patients, clinicians regarded the ePRO system as useful for providing patients with reassurance about how to manage their symptoms appropriately. Additionally, clinicians found the ePRO symptom-reports provided a valuable insight into understanding participants’ experiences of recovery and monitoring symptoms in the context of ongoing recovery. This insight was found to be particularly relevant for facilitating telephone consultations with participants:

*Clinician 2: “I found [the ePRO symptom-report system] particularly useful on the phone because when you’re speaking to a patient on the phone, all the visual cues are lacking… you can run through all of the things you’d normally speak to them about, but then you can also say, oh…your such and such [symptom-report on the ePRO system] was a little bit concerning. And it can help to guide the conversation but, it can also make sure that things aren’t overlooked, because often, particularly in a telephone conversation they can be.”*

The CNS team commented that being able to access ePRO symptom reports via the hospital EHRs was appropriate and useful. However, they acknowledged that use of the ePRO system may be influenced by existing complexities of hospital systems and limited access to computers:

*Clinician 1: “It’s just like in clinic sometimes we can’t always access a computer to look at [patient results] before [patients] come in.”*

### Technical performance

Between 25/08/2017 and 15/03/2018, there was one incident of unplanned downtime between the ePRO system and EHRs which resulted in clinicians being unable to view ePRO symptom reports via EHRs. There was one incident where a participant was unable to access the ePRO system at baseline due to an administrative error. Both issues were resolved within one and 5 days respectively. Due to a University of Bristol IT server update there was a five-day period of downtime in the participant email/text message reminder system. This resulted in four participants having their reminder messages delayed by up to 5 days.

### Piloting of potential outcome measures for a main trial

Completed FACT-G and EQ-5D questionnaires were returned by 19 (66%) and 20 (69%) of participants at week four post-discharge and 15 (52%) at week eight. Fifteen (52%) health resource use forms were returned.

## Discussion

The findings from this pilot study indicate that this novel electronic system for the real-time, remote monitoring of symptoms and problems in patients recovering from cancer-related UGI surgery is feasible and acceptable to both patients and clinicians. Adherence to the routine symptom report completions was good and patients described the ePRO system as reassuring and a valuable support to the self-management of their symptoms, particularly when they were uncertain if those symptoms required clinical intervention. Clinicians regarded the system as a useful adjunct to the routine clinical management of patients. The system detected symptoms indicative of adverse events, including numerous concerning symptoms that prompted contact with HCPs. Appropriate alerts were provided to clinicians and the system identified severe complications associated with hospital re-admissions. The system also worked well in a less-experienced hospital that had not been involved in the developmental work. It is therefore recommended that the ePRO system is further evaluated in the context of a clinical effectiveness study to inform full implementation into the healthcare system.

Studies of other ePRO systems have typically focused on evaluating the feasibility of PRO data collection alone, rather than their real-time integration of ePRO data into routine post-discharge clinical management [41–44]. A recent RCT of 344 patients in the Netherlands concluded that use of a “personalised” e-Health programme improved rates of return to normal activities compared to usual care following general or gynaecological surgery for benign conditions [45]. This programme was not integrated into hospital EHR and alerts to contact a health professional were sent only to patients reporting a delayed recovery, and not to clinicians. Patient feedback was based only on data relating to resumption of daily activities inputted by patients a priori and tailored only to patient demographic factors (e.g. surgical procedure, sex). The nature and severity of symptoms experienced by patients was also not measured. The functionality to provide individually-tailored advice based on symptom severity has been identified as particularly important for the effectiveness of ePRO systems [46–48]. Similarly, the incorporation of clinician alerts to ePRO systems can increase clinician involvement in patient care and improve patient outcomes [25, 29]. However, some clinicians may not act upon alerts generated by symptom-report systems [15], possibly due to perceived disruption to usual workflow pathways [49] or uncertainty over how to respond. To overcome these issues, the ePRO surgery system reported in this study not only alerts clinicians to concerning symptoms but also provides guidance about responding to alerts and has been developed with clinician input and incorporation into existing clinical pathways [21, 50, 51].

ERAS protocols lack standardisation around the timing and nature of clinical follow-up for patients following cancer-related UGI surgery [52], with most clinical contact occurring shortly after hospital discharge. However, in the current study, nearly half of all self-management advice and advice to contact an HCP was triggered in the latter period of recovery, up to 2 months post-discharge. This information is useful to inform assessment time points in a future RCT. It also highlights an unmet healthcare need for the routine monitoring of symptoms and the provision of advice after the acute phase of patients’ recovery has passed. This study supports previous research indicating that it would be beneficial to extend current ERAS protocols to encompass the real-time monitoring of symptoms over a prolonged period post-discharge [53].

In current practice, patients must accurately recall relevant symptom-management information after their discharge from hospital, which is generally delivered verbally prior to treatment [54]. Recall may be impaired by pain, fatigue, sleep deprivation [55] and cognitive dysfunction associated with critical care [56]. ePROM platforms that systematically deliver individually-tailored patient information have been associated with increased information retention [57] and improved patient outcomes [54, 58, 59]. Patients reported that the capacity for the ePRO surgery system to monitor symptoms in real-time and provide individually tailored advice on demand enabled them to proactively and promptly manage their symptoms. The benefit and impact of this can further be investigated within a main trial. In addition, clinicians perceived a key advantage of the ePRO surgery system to be the automated signposting of patients experiencing problems to appropriate sources of healthcare. Clinicians did, however, identify potential barriers to using the ePRO surgery system relating to integration with hospital IT systems and EHR such as periods of system downtime, replicating findings from other studies [42, 49, 50, 60, 61]. For successful implementation in clinical practice, developers of ePRO systems should ensure enough time and resources are dedicated to IT integration and ongoing support of live systems*.* Widespread adoption of hospital-integrated ePRO systems is also dependent upon patients having access to IT resources at home. Approximately one fifth of patients approached to take part in this study were not eligible because they did not have access to a computer, laptop, mobile device or the internet at home. This reflects internet usage trends of UK households of those aged 65 and over [62]. Incorporating electronic systems into usual care pathways will require attention to providing additional monitoring of patients who are unable to use the systems.

The ePRO surgery system has been developed and tested in a diverse group of patients. Mixed methods were used to evaluate system functioning, adherence to the study protocol, quality and completeness of data and patients’ and clinicians’ views towards acceptability of using the system alongside usual care. Symptom self-report items originated or were informed by established, validated measures used widely in studies of cancer patients. Participant refusal rates are in keeping with other similar pilot studies [63] and studies of surgical patients [64–66] and likely reflect in part how unwell participants were feeling soon after major cancer surgery when they were approached about recruitment to the study. It is possible that patients who declined to participate may have been feeling more unwell than those who took part. However, patients must have reached a certain stage in their recovery to have been considered by the healthcare team as fit to be discharged, which is the point at which patients were approached about participation. Furthermore, there did not appear to be any major differences between patients who did and did not take part. The impact of the timing of approaching patients and how to safely monitor patients who decline to use the ePRO system are important points to consider should the system be implemented in routine clinical practice in the future. Self-report completion rates and adherence to the ePRO surgery system protocol were good, demonstrating that weekly completion of the ePRO symptom-report and duration of follow-up period were feasible and acceptable. Participants received weekly and end of study data-collection telephone interview from a researcher. While these calls were not intended to discuss patients’ symptoms or provide advice regarding managing or seeking treatment for problematic symptoms, it is possible that receiving this weekly contact may have influenced ePRO questionnaire completion rates*.* While several participants withdrew from the study because they felt too unwell, this was expected due to the nature of the patient group and the major surgery they had experienced. Data completion was lowest at the earliest timepoint post-discharge when patients were likely to be experiencing the most frequent and severe symptoms. It is important that consideration is given to instances when patients feel too unwell to access the system. It may therefore be beneficial to incorporate a mechanism in the system whereby non-response at critical time points is regarded as an indicator of potentially concerning symptoms and a clinician alert is triggered. Clinicians at Centre 2 were unable to access patients’ symptom reports via EHRs, preventing pooling of data with that from Centre 1, which may have limited clinicians’ engagement with the ePRO system. There are also further refinements that may improve the system’s performance, including removing the pre-discharge Level 3 alert for symptoms indicative of a complication, and amending the wording of symptom-report items to ensure symptoms that have already been resolved or are due to unrelated underlying conditions do not trigger clinician alerts.

## Conclusion

A real-time, hospital-integrated symptom monitoring system has been developed to optimise the prompt and effective management of symptoms and complications experienced by patients after discharge following abdominal cancer-related surgery. Following the findings from this pilot study indicating that the ePRO surgery system is feasible and acceptable to patients and clinicians, a definitive RCT to evaluate the impact of the system on patients’ physical wellbeing is warranted.

## Supplementary information

**Additional file 1.** Interview Topic Guides.

## Data Availability

The datasets used and/or analysed during the current study are available from the corresponding author on reasonable request. Full interview transcripts are not available to protect participant anonymity.

## Abbreviations

CNS: Cancer nurse specialists
EHR: Electronic health record(s)
EORTC: European Organisation for Research and Treatment of Cancer questionnaire
ePRO: Electronic patient reported outcome
ePROM: Electronic patient reported outcome measure
eRAPID: Electronic patient self-Reporting of Adverse-events: Patient Information and aDvice
ERAS: Enhanced recovery after surgery
GP: General practitioner
HCP: Healthcare professional
HRQL: Health-related quality of life
NHS: National Health Service
PRO: Patient-reported outcome
PROM: Patient-reported outcome measure
RCT: Randomised controlled trial
SD: Standard deviation
UGI: Upper gastrointestinal
